# Substituent Flexibility Modulates Aggregation-Induced Emission in Tetraphenylbenzene

**DOI:** 10.1021/acs.orglett.5c02947

**Published:** 2025-09-02

**Authors:** Vincent Monnier, Federico Begato, Aurelio Mateo-Alonso

**Affiliations:** a POLYMAT, 83067 University of the Basque Country UPV/EHU. Avenida de Tolosa 72, 20018 Donostia-San Sebastian, Spain; b Ikerbasque, Basque Foundation for Science, 48009 Bilbao, Spain

## Abstract

Among the existing aggregation-induced emission luminogen archetypes, tetraphenylbenzene is one of the simplest and less studied for its aggregation-induced emission properties. In this study, we give insight into the structure–AIE relationship, focusing on the functionalization of the two free *para* positions of the benzene core of tetraphenylbenzene. Our study reveals that the flexibility and rigidity of the *para* substituents noticeably impact the aggregation-induced emission expression, allowing the inducement of either aggregation-induced emission enhancement or pure aggregation-induced emission.

## Graphical abstract

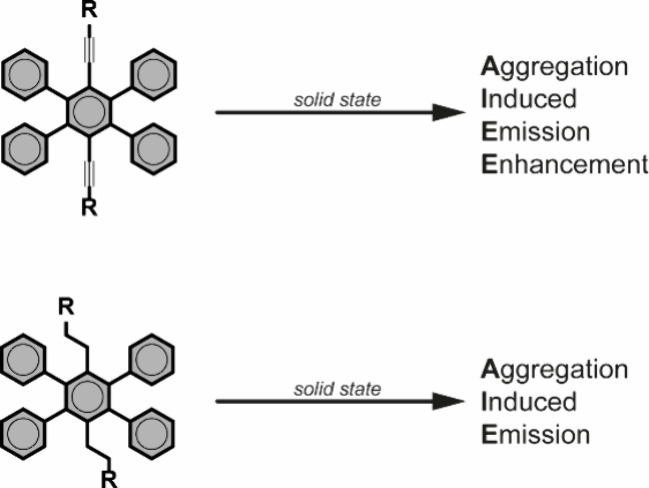

Aggregation-induced emission (AIE) is a photophysical phenomenon through which certain molecules, which are nonemissive or weakly emissive in solution, present a turn-on (pure AIE) or strong increase (AIEE) of luminescence as they aggregate in solution or in the solid state.^1,2^ This phenomenon is highly relevant in several applications, such as sensing^3–8^ or solid-state electroluminescent devices,^9–11^ where solid-state luminescence is needed.

Generally, the AIE mechanism is considered to evolve from the restriction of intramolecular rotations in the aggregated state, implying a suppression/diminution of nonradiative relaxation pathways of the excited state.^2^ Good performing aggregation-induced emission luminogens (AIEgens) are often molecules that present conjugated moieties, typically phenyls, connected together through simple σ-bonds, allowing for their rotation in the solvated state (quenched state) that would be frozen in the solid state (emissive state). Famous archetypes of single small molecule AIEgens include tetraphenylethene (TPE)^12–21^ and hexaphenylbenzene (HPB) moieties,^22–28^ sometimes even both,^7,29^ at the core of their molecular design.

A simpler parent of HPB is 1,2,4,5-tetraphenylbenzene (TPB), where the phenyl groups in two *para* positions are exchanged for other substituents (see Figure 1). This motif presents a noteworthy difference with HPB, or even TPE, when it comes to the design of AIEgen: due to their proximity to the rotating phenyls, the free *para* positions of the benzene core allow substituents to directly impact the AIE properties. However, despite its synthetic accessibility, the TPB motif has been only scarcely explored comparatively to HPB.^9,30^

**Figure 1:**
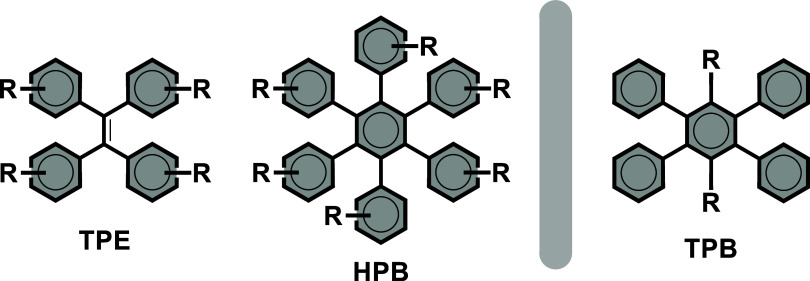
Structures of the well-known TPE and HPB AIEgens and of the TPB AIEgens studied here.

In this work, we show how the conformational flexibility and the bulkiness of the *para* substituents of TPB derivatives impact their AIE expression, giving rise to aggregation-induced emission enhancement (AIEE) in the case of rigid substituents or to pure AIE in the case of conformationally flexible substituents.

In order to investigate the effects of bulkiness and flexibility on the properties of TPB AIEgens, two different types of *para* substituents were first introduced on the bromanil core **1** by adapting a known route^31,32^ that involves a two-step reductive alkynylation procedure. This adapted method afforded derivatives **2** and **3**, when using TIPS-acetylene and 1-octyne, respectively (Scheme 1). Subsequently, a 4-fold Suzuki cross-coupling with phenylboronic acid allowed us to reach the target rigid derivatives **TPB-TIPS-acetylene (4)** and **TPB-octyne (5)**. Finally, reduction of the rigid alkyne by catalytic hydrogenation afforded **TPB-TIPS-ethylene (6)** and **TPB-octyl (7)** with conformationally flexible ethylene groups.

**Scheme 1:**
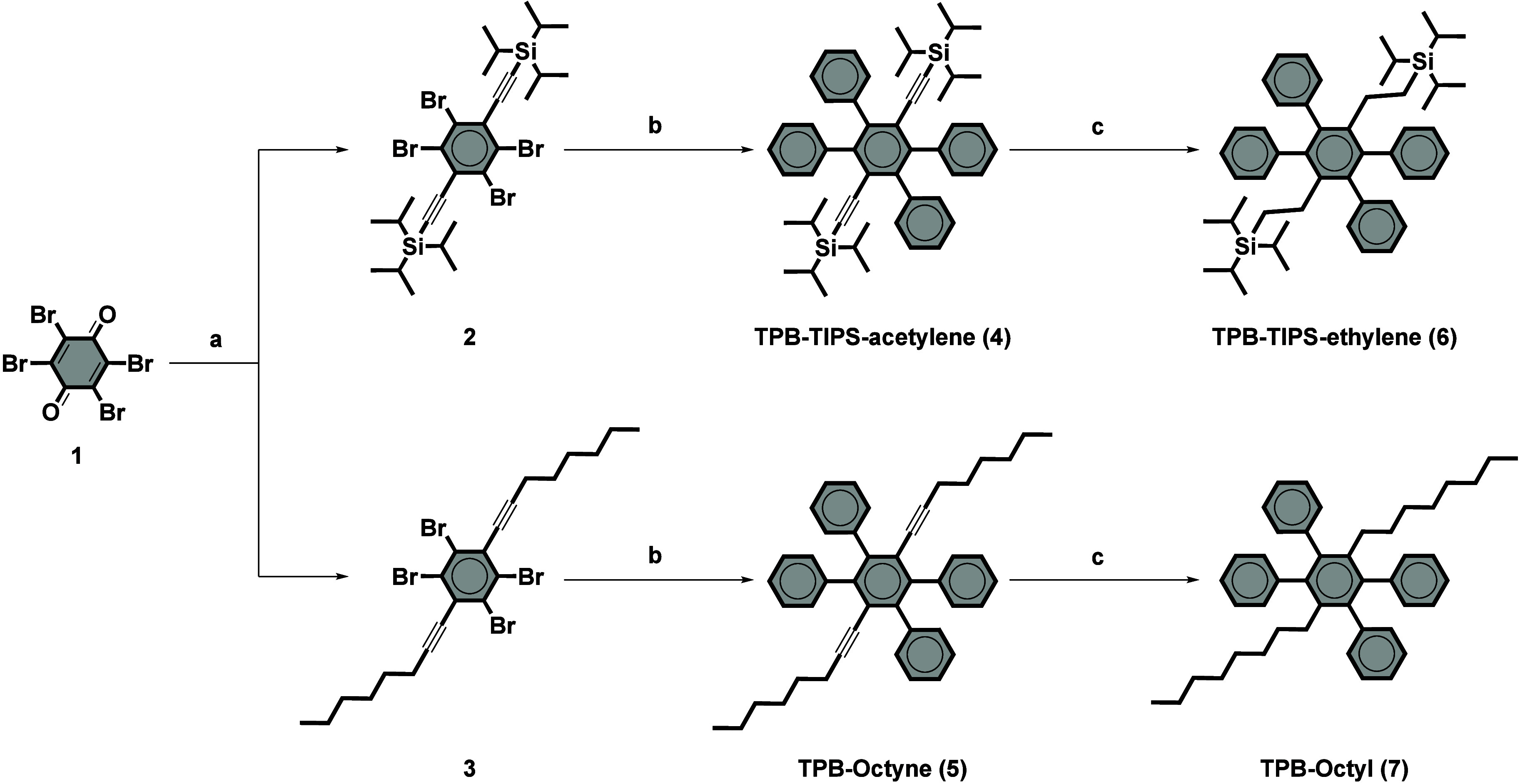
Synthesis of Four Derivatives of TPB, Bearing *para* Substituents of Various Rigidities and Bulkiness^a^

a (a) 1) H-alkyne, *n*-BuLi, (THF), 25 °C → 60 °C, 1 h, 2) SnCl_2_, (CH_3_CN/H_2_O), 90 °C, 12 h, [**2**: 76%], [**3**: 54%]; (b) Phenylboronic acid, [Pd­(PPh_3_)_4_], Cs_2_CO_3_, (Dioxane/H_2_O), 90 °C, 19 h, [**TPB-TIPS-acetylene (4)**: 78%], [**TPB-octyne (5)**: 77%]; (c) H_2_, Pd/C, (Hexane/Butanol), 25 °C, 19 h, [**TPB-TIPS-ethylene (6)**: 82%], [**TPB-octyl (7)**: 90%].

The four target compounds **TPB-TIPS-acetylene (4)**, **TPB-TIPS-ethylene (6)**, **TPB-octyne (5)**, and **TPB-octyl (7)** are colorless, absorbing in the UV region (Figure 2). The alkyne-bearing derivatives **TPB-TIPS-acetylene (4)** and **TPB-octyne (5)** present a more red-shifted absorption, extending to 325 nm due to the conjugation of their triple bonds with the π-system. The UV–vis absorption spectrum of **TPB-TIPS-acetylene (4)** is slightly more red-shifted than its alkyl homologue **TPB-octyne (5)**, presenting a lowest energy maximum of absorption at λ_max_(**TPB-TIPS-acetylene (4)**) = 310 nm, while λ_max_(**TPB-octyne (5)**) = 297 nm in THF. The reduced **TPB-TIPS-ethylene (6)** and **TPB-octyl (7)** derivatives present two identical absorption spectra, as the conjugation extends on identical tetraphenylbenzene systems, while the lateral TIPS or alkyl chains can only influence the electronic properties via inductive effects, which appear to be negligible.

The emission properties in solvated state (Figure 2) were carried out in THF, as it allows the subsequent AIE study by addition of water.^30,33^ Compounds **TPB-TIPS-acetylene (4)** and **TPB-octyne (5)** are emissive in the solvated state. Comparatively, derivatives **TPB-TIPS-ethylene (6)** and **TPB-octyl (7)** are virtually not emissive.

**Figure 2:**
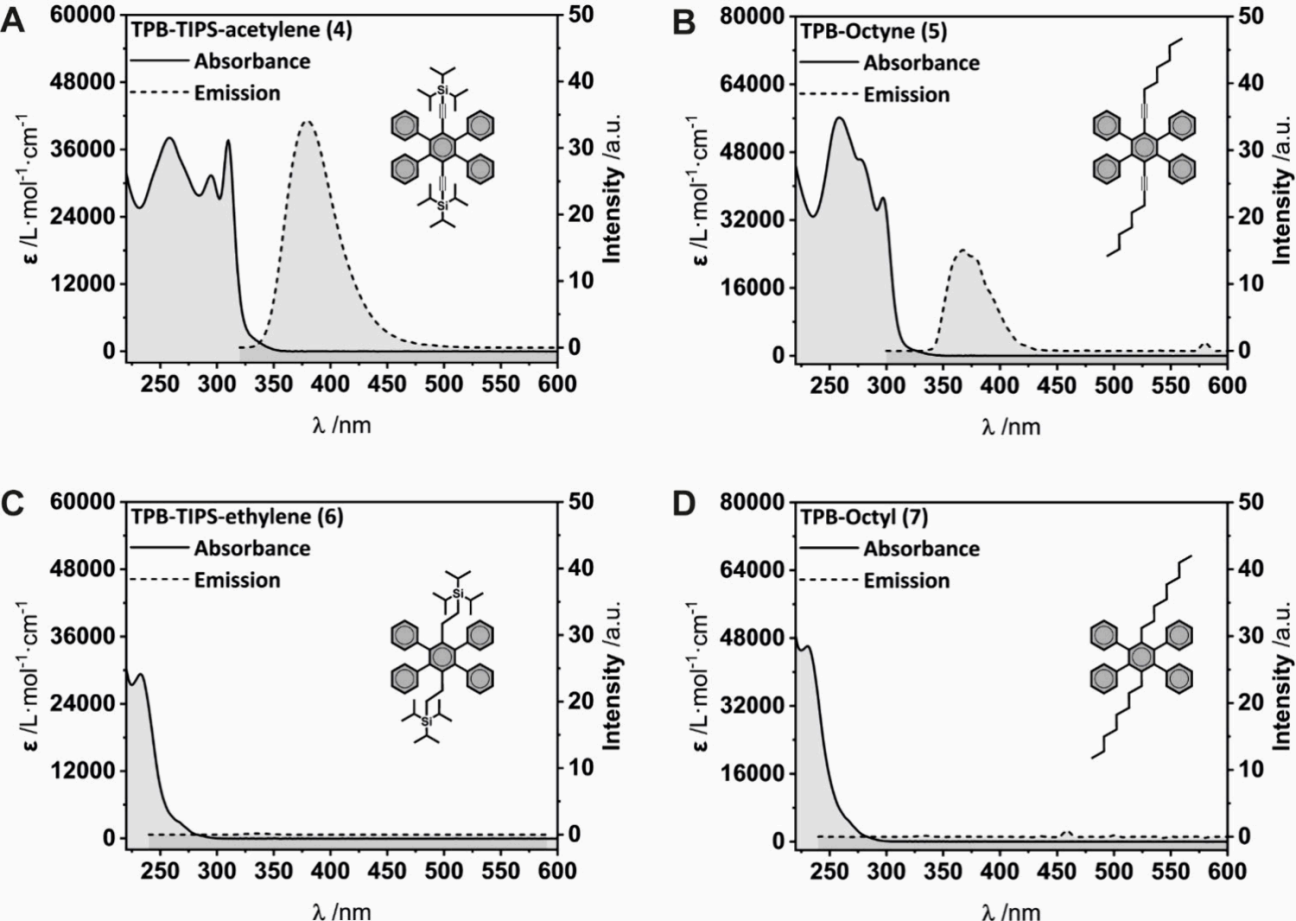
Molar absorptivity (left, solid line) and relative emission intensity of A) **TPB-TIPS-acetylene (4)**, B) **TPB-octyne (5)**, C) **TPB-TIPS-ethylene (6)** and D) **TPB-octyl (7)**. Measurements were performed in THF.

In the context of simple fluorophores such as BTP, presenting very similar polarities and in the absence of additional effects such as charge transfer or excimer formations, Stokes shifts can be used as indirect tools to quantify the relative conformational flexibility.^34–37^ The higher the shift between absorption and emission, the higher the flexibility in the excited state and as such the higher the flexibility in the fundamental state. It should hence be possible to observe correlation tendencies between higher Stokes shifts and lower emitters as more flexibility implies more vibrational relaxation pathways for the excited states. **TPB-octyne (5)** (Δν = 6496 cm^–1^) displays a larger Stokes shift when compared to **TPB-TIPS-acetylene (4)** (Δν = 5942 cm^–1^); this is consistent with the higher flexibility of the octyne group.

AIE was investigated by measuring the emission of solutions and suspensions of target molecules in different THF/H_2_O mixtures (Figure 3). These measurements were performed at a constant concentration chosen for both (i) aggregation to occur in the range of THF/H_2_O ratios and (ii) for a similar value of absorbance at the excitation wavelength for each of the studied derivatives (ca. 0.3 mM, see the Supporting Information). Our study reveals that the four targeted compounds present AIE behavior. An increase of the signal intensity is detected as soon as the fraction of water is sufficient to induce the formation of aggregates/precipitates (Figure 3). On one hand, **TPB-TIPS-acetylene (4)** and **TPB-octyne (5)** behave as aggregation-induced emission enhancement luminogens (AIEEgens),^5,8,14,38^ as they weakly emit in THF. On the other hand, **TPB-TIPS-ethylene (6)** and **TPB-octyl (7)** behave as pure AIEgens, presenting a complete turn-on/turn-off of their luminescence, consistent with previous observations on tetraphenylbenzene^39,40^ and tetraphenylpyrazine^41^ derivatives.

**Figure 3:**
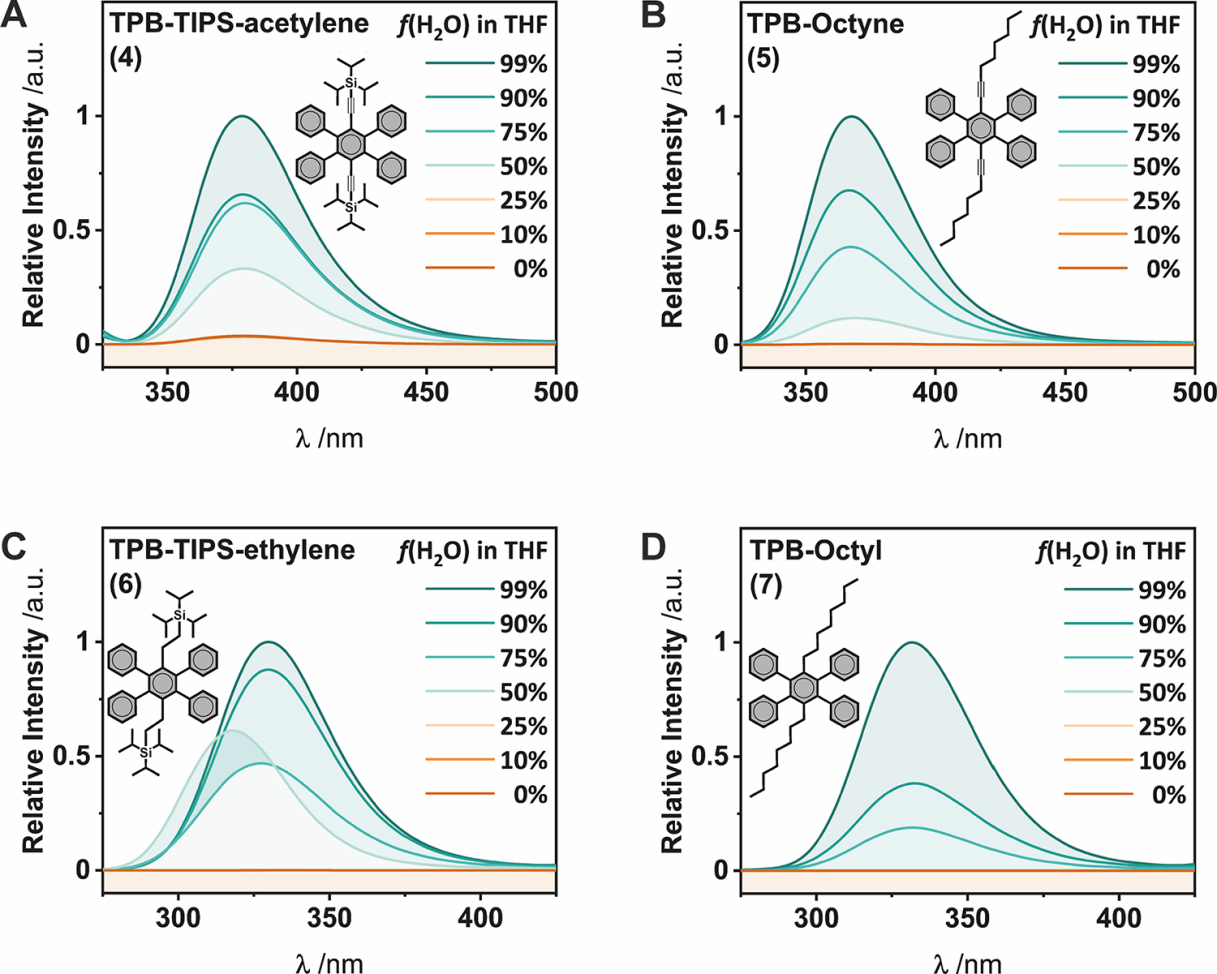
Emission spectra of equimolar solutions of A) **TPB-TIPS-acetylene (4)**, B) **TPB-octyne (5)**, C) **TPB-TIPS-ethylene (6)** and D) **TPB-octyl (7)** with different percentages of H_2_O in THF.

Interestingly, while the luminescence intensity increases significantly, no energy shift is observed, except for the trace at 50% H_2_O for TPB-TIPS-ethylene, which appears blue-shifted. However, this deviation is not persistent and is not observed at different H_2_O percentages. This suggests that, although intermolecular interactions are necessarily present in the aggregated state, these AIEgens do not form distinct H- or J-aggregates that would significantly alter the energies of the electronic states. This allows the Stokes shifts of the alkyl AIEgens to be determined, and that shows again a larger Stokes shift for **TPB-octyl (7)** (Δν = 13170 cm^–1^) when compared to **TPB-TIPS-ethylene (6)** (Δν = 12625 cm^–1^), consistent with the higher flexibility of the octyl chains.

The quantum yields of luminescence of these compounds have been determined indirectly, using 9,10-diphenylanthracene as a reference, for fractions of water of *f*(H_2_O) = 0% and *f*(H_2_O) = 99% (see Table 1). A drastic overall increase of all quantum yields of luminescence is observed for each compound, confirming what is qualitatively approached in Figure 3.

**Table 1:** Optoelectronic Characterization

	**λ**_**max**_ **(abs)**	**λ**_**max**_ **(em)**	**Δν**	**ε**_**λ_max_**_	**Φ**_**em**_	**Φ**_**em**_
	nm	nm	cm^–1^	L·mol^–1^·cm^–1^	*f*(H_2_O) = 0%	*f*(H_2_O) = 99%
**TPB-TIPS-acetylene (4)**	310	380^a^	5942	37650	46	69^b^
**TPB-octyne (5)**	297	368^a^	6496	37300	3	23^b^
**TPB-TIPS-ethylene (6)**	233	330^a^ ^,^ ^b^	12625	29300	0	19^b^
**TPB-octyl (7)**	231	332^a^ ^,^ ^b^	13170	46100	0	20^b^

a Excitation wavelengths: λ_exc_(**TPB-TIPS-acetylene (4)**) = 315 nm; λ_exc_(**TPB-octyne (5)**) = 297 nm; λ_exc_(**TPB-TIPS-ethylene (6)**) = 232 nm; λ_exc_(**TPB-octyl (7)**) = 230 nm. All results were measured in THF except where marked with *b*.

b Measured in THF/H_2_O 1:99.

In conclusion, four TPB derivative cores have been synthesized. Their UV–vis absorption and emission properties have been investigated as well as their AIE behavior. This study revealed that the derivatives including a triple bond, namely, **TPB-TIPS-acetylene (4)** and **TPB-octyne (5)**, are photoluminecent in solution and their luminescence increases in the aggregated state, illustrating their AIEE behavior. On the other hand, TPB derivatives with more flexible alkyl chains, namely, **TPB-TIPS-ethylene (6)** and **TPB-octyl (7)**, do not show any detectable luminescence in solution, whereas their emission is turned on in the aggregated state, illustrating their purely AIE behavior. This study shines additional light on the impact of *para*-substituents on the AIE behavior of TPB, offering insights that may guide the design of advanced AIEgens with tunable and enhanced emission characteristics.

## Supplementary Material



## Data Availability

The data underlying this study are available in the published article and its Supporting Information.

## Notes

Experimental details and characterization data of all the products, including NMR and MS spectra (PDF)
