# Supplementary material for: Substituent Flexibility Modulates Aggregation-Induced Emission in Tetraphenylbenzene
Source: Org Lett. 2025 Sep 2;27(36):9976–81. doi: 10.1021/acs.orglett.5c02947 (PMC12442222; doi:10.1021/acs.orglett.5c02947)
Supplement: Supplementary file 1 [file ol5c02947_si_001.pdf]

Supplementary information for:

Substituent Flexibility Modulates Aggregation-Induced Emission in Tetraphenylbenzene

Vincent Monnier[a], Federico Begato[a] and Aurelio Mateo-Alonso[a,b]*

[a] POLYMAT, University of the Basque Country UPV/EHU. Avenida de Tolosa 72, 20018 Donostia-San Sebastian (Spain)

[b] Ikerbasque, Basque Foundation for Science, 48009 Bilbao (Spain)

Contents

General Information 3
Synthesis protocols 5
NMR spectra 10
MS spectra 16
IR spectra 19
Emission and Excitation spectra in pure THF 20
Aggregation Induced Emission 21
Electrochemical characterization 23
References 25

General Information

Commercially available solvents and reagents were used without further purification unless otherwise noted. Column chromatography was carried out using a Silica gel 60 from Scharlab.

^{1}H and ^{13}C NMR spectra were recorded on a Bruker Avance 400 spectrometer at 298 K using partially deuterated solvents as internal standards.

High-resolution mass spectrum of compound **3** was recorded by Dr. Javier Calvo on UltrafleXtreme III MALDI tandem mass spectrometer (Bruker) in reflector acquisition operation mode and the sample was prepared in CH_2Cl_2. High-resolution mass spectra of compounds **TPB-TIPS-acetylene (4)**, **TPB-Octyne (5)**, **TPB-TIPS-ethylene (6)**, and **TPB-Octyl (7)** were recorded by Dr. Dorleta Otaegui at POLYMAT on a Daltonics-Autoflex MALDI-TOF mass spectrometer (Bruker) in reflector acquisition operation mode and the samples were prepared in CH_2Cl_2.

UV/visible absorption spectra were recorded on a Perkin-Elmer Lambda 950 spectrometer.

Fluorescence spectra were registered on a LS55 Perkin-Elmer Fluorescence spectrometer. Fluorescence quantum yields were determined using an external reference, 9,10-diphenylanthracene (DPA), in solution in cyclohexane, for which the quantum yield is set at $\Phi_{DPA, cyclohexane} = 1$.

Titration experiments were performed by preparing 10 mL solutions of the proper THF/H_2O ratio, minus 0.1 mL of THF. To these solutions, 0.1 mL of a mother 1 mL THF solution containing 10 times the desired amount of analyte was added vigorously in one portion, inducing direct mixing (and precipitation if it had to occur) of the analyte. The used concentrations for the analytes were aimed to i) induce a precipitation at a reasonably low water fraction ($f(H_2O)$ 50%-75%) and ii)

present ideally an identical $A_{\lambda max}$. The used concentrations were hence: [1b] = 0.28 mM ; [2b] = 0.35 mM ; [1c] = 0.25 mM ; [2c] = 0.22 mM. The refractive indexes of the solvents were determined from the fitting of a part of a THF/water mixture RI measurement from literature.[1] The value obtained for a 1:99 THF/H_2O mixture was 1.3355.

Fourier transform infrared (FT-IR) spectra were taken on a Bruker Optics ALPHA-E spectrometer with a universal Zn-Se ATR (attenuated total reflection) accessory in the 400–4000 cm^{-1} region or using a Diamond ATR (Golden Gate).

Cyclic voltammetry. Electrochemical behavior of the compounds was studied at room temperature using cyclic voltammetry in a three-electrode single-compartment cell consisting of a glassy carbon disk (3 mm diameter), working electrode silver wire as the reference electrode, and a platinum wire as the counter electrode with ferrocene (Fc) as an internal reference (0.3 mM). The GC electrode surface was cleaned by polishing with a 0.05 μm Alumina MicroPolish powder prior to each experiment, followed by ultrasonically rinsing in ethanol for 5 min. The cell was connected to the computer controlled potentiostat (Princeton Applied Research - PARSTAT 2273). The measurements were carried out under Ar atmosphere in anhydrous THF using tetrabutylammonium hexafluorophosphate (0.1 M) as the supporting electrolyte. The concentration of the prepared samples was in the range of 1–2 mM. Initial potential 0 V, reductive direction of initial scan, switching potentials -2.5 V and 1.6 V, scan rate 100 mV/s.

Synthesis protocols

Scheme 1. Synthesis of four derivatives of **TPB**, bearing *para* substituents of various rigidities and bulkiness.[a]

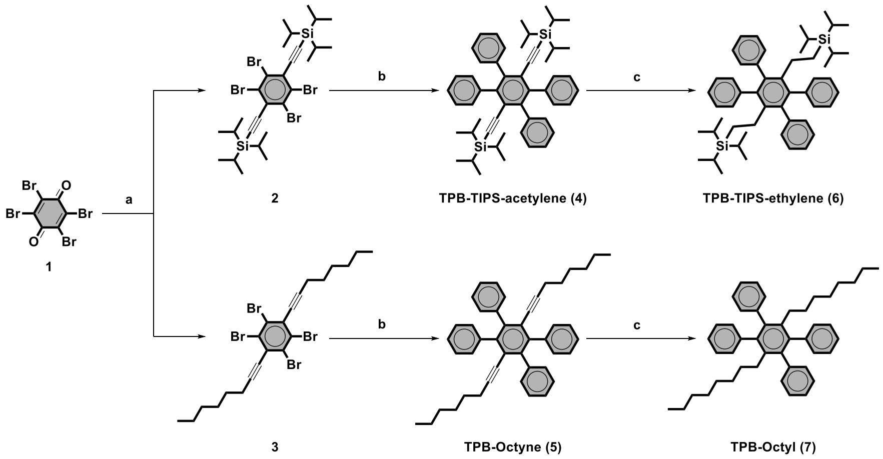

[a] (a) 1) H-alkyne, *n*-BuLi, (THF), 25 °C → 60 °C, 1 h, 2) $SnCl_2$, (CH_3CN/H_2O), 90 °C, 12h, [**2**: 76%], [**3**: 54%]; (b) Phenylboronic acid, [$Pd(PPh_3)_4$], Cs_2CO_3, (Dioxane/H_2O), 90 °C, 19 h, [**TPB-TIPS-acetylene (4)**: 78%], [**TPB-Octyne (5)**: 77%]; (c) H_2, Pd/C, (Hexane/Butanol), 25 °C, 19 h, [**TPB-TIPS-ethylene (6)**: 82%], [**TPB-Octyl (7)**: 90%]

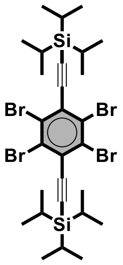

2

2 was synthesized according to literature procedure.[2] TIPS-acetylene (609 µL, 2.715 mmol) was dissolved in dry THF (9.5 mL) at 25 °C, and *n*-BuLi (2.5 M in Hexanes, 1.04 mL, 2.6 mmol) was added dropwise and the medium was stirred for 30 min. Bromanil **1** (500 mg, 423.68 g/mol, 1.18 mmol) was added in one portion and the medium was stirred at 60 °C using an oil bath for 30 min. Eventually, NH_4Cl aq. sat. was added (10 mL) and the products were extracted with Hexane (1x) and with DCM (2x). The organic phases were concentrated *in vacuo*, and the dry crude was dispersed/dissolved in ACN (25 mL) and H_2O (0.1 mL), added of $SnCl_2$ (900 mg) and refluxed using an oil bath for 12 h. Eventually, the product is recovered by filtration affording **2** as a white solid (682 mg, 754.43 g/mol, 76%).

^{1}H NMR ($CDCl_3$, 400 MHz): *partially merged* [1.16+1.15] (42H, m).

^{13}C NMR ($CDCl_3$, 100 MHz): δ 128.6, 107.0, 104.9, 18.7, 12.6.

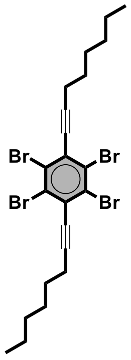

3

1-Octyne (400 µL, 2.715 mmol) was dissolved in dry THF (9.5 mL) at 25 °C, and *n*-BuLi (2.5 M in Hexanes, 1.04 mL, 2.6 mmol) was added dropwise and the medium was stirred for 30 min. Bromanil **1** (500 mg, 423.68 g/mol, 1.18 mmol) was added in one quick portion and the medium was stirred at 60 °C using an oil bath for 30 min. Eventually, NH_4Cl aq. sat. was added (10 mL) and the products were extracted with hexane (1x) and with dichloromethane (2x). The organic phases were concentrated *in vacuo*, and the dry crude was dispersed/dissolved in acetonitrile (25 mL) and H_2O (0.1 mL), added of $SnCl_2$ (900 mg) and refluxed using an oil bath for 12 h. Eventually, the product is recovered by filtration affording **3** as a white shiny powder (380 mg, 610.07 g/mol, 54 %).

^{1}H NMR ($CDCl_3$, 400 MHz): δ 2.56 (4H, t, J_1=6.54 Hz), 1.70-1.63 (4H, m), 1.53-1.48 (4H, m), 1.36-1.30 (8H, m), 0.90 (6H, t, J_1=7.34 Hz).

^{13}C NMR ($CDCl_3$, 101 MHz): δ 129.1, 127.9, 103.4, 81.2, 31.5, 28.7, 28.3, 22.7, 20.0, 14.2.

HRMS (ESI-TOF) m/z: [M + Ag]+ Calcd for $C_{22}H_{26}Br_4Ag$ 712.7819; Found 712.7790.

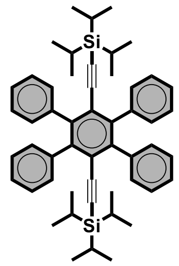

TPB-TIPS-acetylene (4)

2 (300 mg, 754.43 g/mol, 0.398 mmol) and phenylboronic acid (426 mg, 121.93 g/mol, 3.49 mmol, 8.8 eq) were dissolved in a mixture of dioxane/water (9:1, 15 mL) at 25 °C and the atmosphere was purged with argon for 30 min. Eventually, Tetrakis(triphenylphosphine)palladium(0) (183 mg, 1155.54 g/mol, 0.158 mmol, 40%) and cesium carbonate (1.27 g, 325.82 g/mol, 3.9 mmol, 9.8 eq) were added quickly and the medium was purged with argon for 20 min. The solution was then heated at 90 °C using an oil bath under argon for 19 h. The mixture was cooled down to 25 °C and diluted with ethyl acetate. The resulting organic solution was washed with water (2x) and brine (1x). The organic phase was then dried with Na_2SO_4 and solvents were evacuated. The title product was isolated with a column chromatography (SiO_2,

Hexane 100% → hexane/ethyl acetate 95:5) and a re-precipitation by slow evaporation (*in vacuo*, 20 °C) from a mixture of dichloromethane and methanol, affording **TPB-TIPS-acetylene (4)** as white crystals (230mg, 743.24 g/mol, 78%).

^{1}H NMR ($CDCl_3$, 400 MHz): δ 7.16-7.03 (20H, m), *partially merged* [0.68+0.67] (42H, s+s).

^{13}C NMR ($CDCl_3$, 101 MHz): δ 143.9, 139.9, 130.6, 127.4, 126.6, 123.2, 105.3, 100.6, 18.4, 11.0.

HRMS (ESI-TOF) m/z: [M + Ag]+ Calcd for $C_{52}H_{62}Si_2Ag$ 849.3441; Found 849.3443.

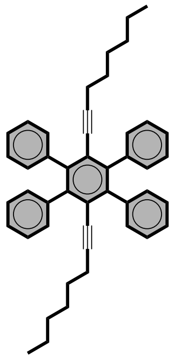

TPB-Octyne (5)

3 (300 mg, 610.07 g/mol, 0.492 mmol) and phenylboronic acid (528 mg, 121.93 g/mol, 4.33 mmol, 8.8 eq) were dissolved in a mixture of dioxane/water (9:1, 15 mL) at 25 °C and the atmosphere was purged with argon for 30min. Eventually, Tetrakis(triphenylphosphine)palladium(0) (227 mg, 1155.54 g/mol, 0.197 mmol, 40%) and cesium carbonate (1.57 g, 325.82 g/mol, 4.82 mmol, 9.8 eq) were added quickly and the medium was sparged with argon for 20 min. The solution was then heated at 90 °C using an oil bath under argon for 19h. The medium was cooled down to 25 °C and diluted with AcOEt. The resulting organic solution was washed with water (2x) and brine (1x). The organic phase was then dried with Na_2SO_4 and solvents were evacuated. The title product was isolated with a column chromatography (SiO2, hexane 100% → Hexane/ethyl acetate 95:5) and a re-precipitation by slow evaporation (*in vacuo*, 20 °C) from a mixture of dichloromethane and methanol, affording **TPB-Octyne (5)** as white crystals (227 mg, 598.87 g/mol, 77%).

^{1}H NMR ($CDCl_3$, 400 MHz): δ 7.17-7.06 (20H, m), 1.85 (4H, t, J_1=6.73 Hz), 1.21-1.12 (4H, m), 1.06-0.97 (8H, m), 0.93-0.88 (4H, m), 0.83 (6H, t, J_1=7.22 Hz).

^{13}C NMR ($CDCl_3$, 101 MHz): δ 143.1, 140.4, 130.8, 127.1, 126.4, 123.2, 99.5, 79.9, 31.5, 28.3, 28.1, 22.6, 19.5, 14.2.

HRMS (ESI-TOF) m/z: [M + Ag]+ Calcd for $C_{46}H_{46}Ag$ 705.2650; Found 705.2655.

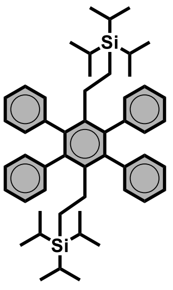
TPB-TIPS-ethylene (6)

TPB-TIPS-acetylene (4) (180 mg, 743.24 g/mol, 0.242 mmol) was dissolved in a mixture of butanol/hexane (1:2, 30 mL) at 25°C and the atmosphere was purged copiously with argon for 30 min. To the medium was then quickly added palladium on carbon (10% Pd) (36 mg, 20% in weight) and the atmosphere was exchanged again for argon through 3 vacuum/argon cycles. The atmosphere was similarly changed for hydrogen and the reacting medium was stirred at 25 °C for 19 h. Once complete conversion of the starting material was observed, the dispersion was filtered through a celite pad (hexane washes). Hexane was removed *in vacuo* and the medium was diluted with methanol, precipitating the compounds as white solids. A silica pad was used to further remove polar impurities (SiO_2, hexane 100%) and the title compound was re-precipitated from a mixture of dichloromethane/methanol by slow evaporation (*in vacuo*, 25 °C), affording **TPB-TIPS-ethylene (6)** (150 mg, 751.30 g/mol, 82%).

^{1}H NMR ($CDCl_3$, 400 MHz): δ 7.11-7.06 (16H, m), 7.01-6.96 (4H, m), 2.41-2.36 (4H, m), 0.56 (42H, bs), 0.49-0.44 (4H, m).

^{13}C NMR ($CDCl_3$, 101 MHz): δ 141.2, 140.8, 139.0, 130.8, 127.4, 125.9, 26.2, 18.4, 11.5, 10.7.

HRMS (ESI-TOF) m/z: [M + Ag]+ Calcd for $C_{52}H_{70}Si_2Ag$ 857.4067; Found 857.4076.

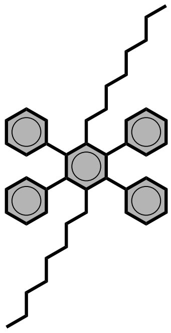

TPB-Octyl (7)

TPB-Octyne (5) (200 mg, 598.87 g/mol, 0.334 mmol) was dissolved in a mixture of butanol/hexane (1:2, 45 mL) at 25 °C and the atmosphere was purged copiously with argon for 30 min. To the mixture was then quickly added palladium on carbon (10% Pd) (40 mg, 20% in weight) and the atmosphere was exchanged again for argon through 3 vacuum/argon cycles. The inert atmosphere was similarly exchanged for hydrogen and the reacting medium was stirred at 25 °C for 19 h. Once the complete conversion of the starting material was observed and the medium was filtered through a celite pad (hexane washes). The hexane was removed *in vacuo* and the mixture was diluted with methanol, precipitating the compounds as white solids. A silica pad was used to further remove polar impurities (SiO_2, Hexane 100%) and the title compound was re-precipitated from a mixture of dichloromethane/methanol by slow evaporation (*in vacuo*, 25 °C), affording **TPB-Octyl (7)** (182 mg, 606.94 g/mol, 90%).

^{1}H NMR ($CDCl_3$, 400 MHz): δ 7.13-7.01 (20H, m), 2.18-2.14 (4H, m), 1.18-1.08 (8H, m), 1.05-0.97 (4H, m), 0.94-0.87 (4H, m), 0.80 (6H, t, J_1=7.28 Hz), 0.76-0.66 (8H, m).

^{13}C NMR ($CDCl_3$, 101 MHz): δ 141.3, 141.0, 136.6, 130.7, 127.1, 125.8, 31.8, 31.5, 30.7, 29.7, 28.9, 28.6, 22.7, 14.2.

HRMS (ESI-TOF) m/z: [M + Ag]+ Calcd for $C_{46}H_{54}Ag$ 713.3276; Found 713.3224.

NMR spectra

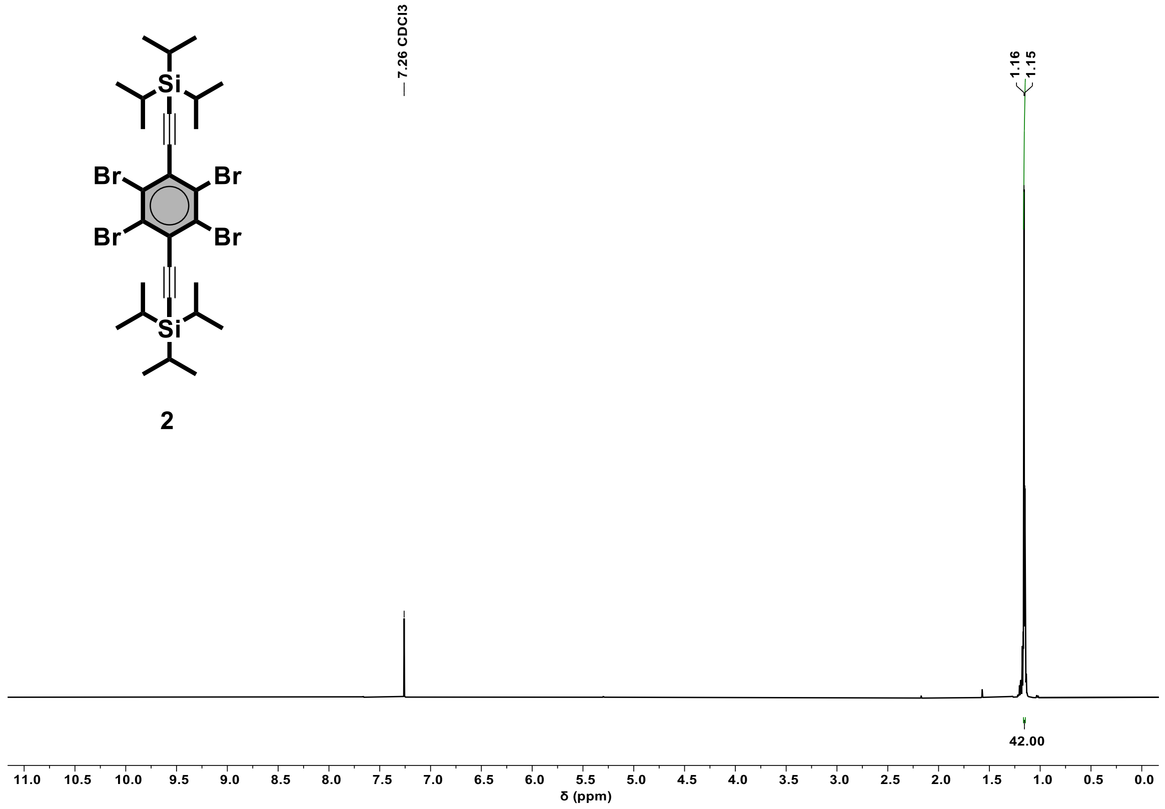

Figure S1: ^{1}H spectrum ($CDCl_3$, 400 MHz) of **2**.

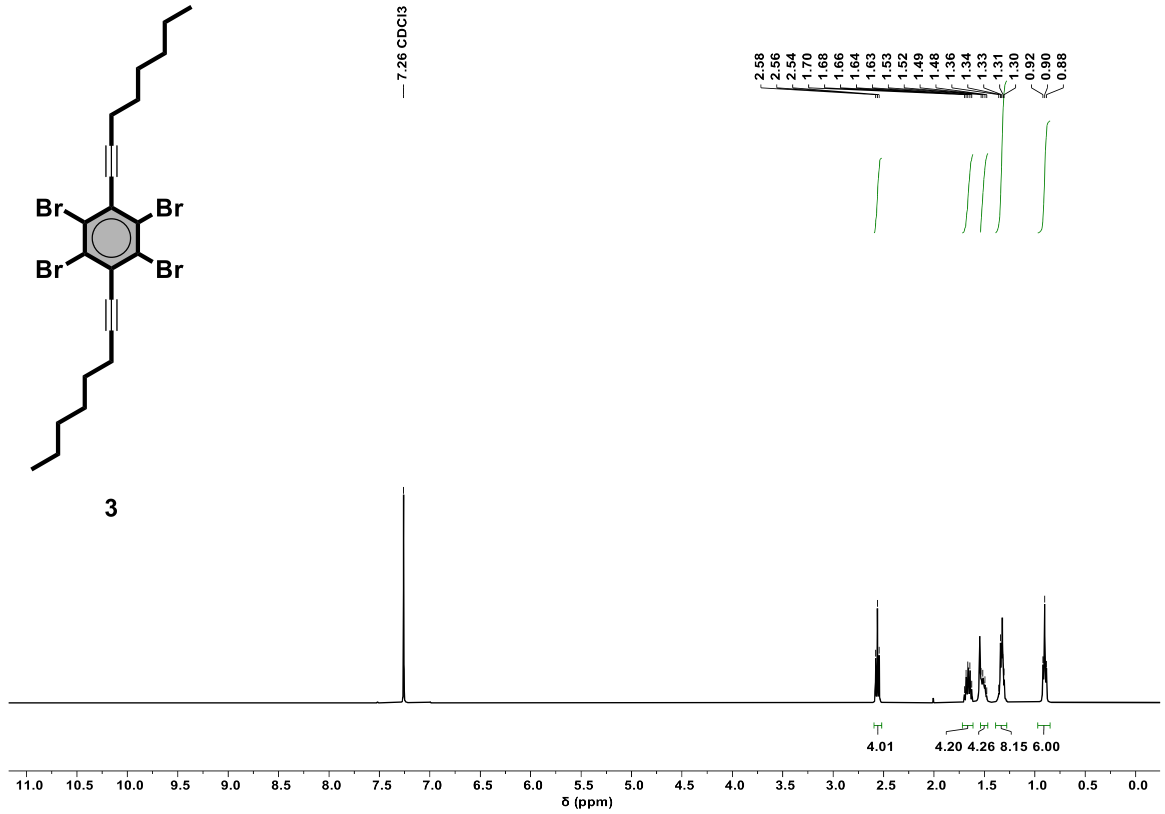

Figure S2: ^{1}H spectrum ($CDCl_3$, 400 MHz) of **3**.

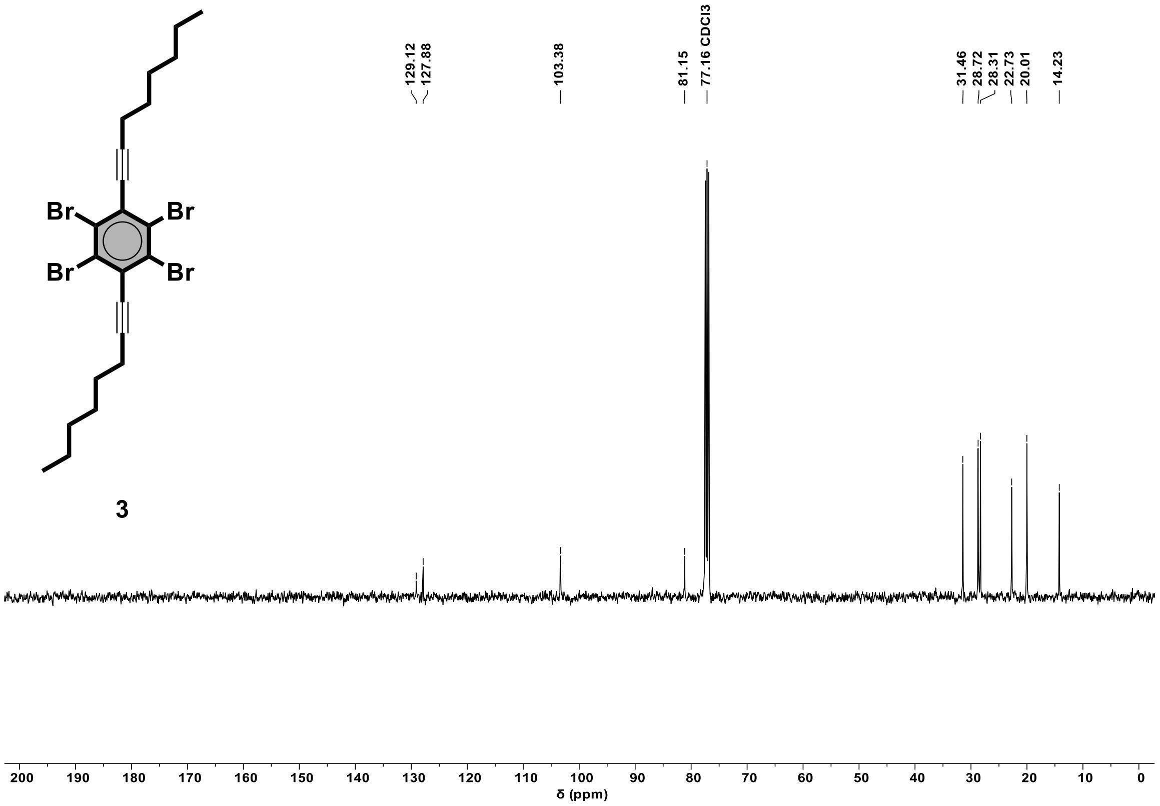

Figure S3: ^{13}C spectrum ($CDCl_3$, 101 MHz) of **3**.

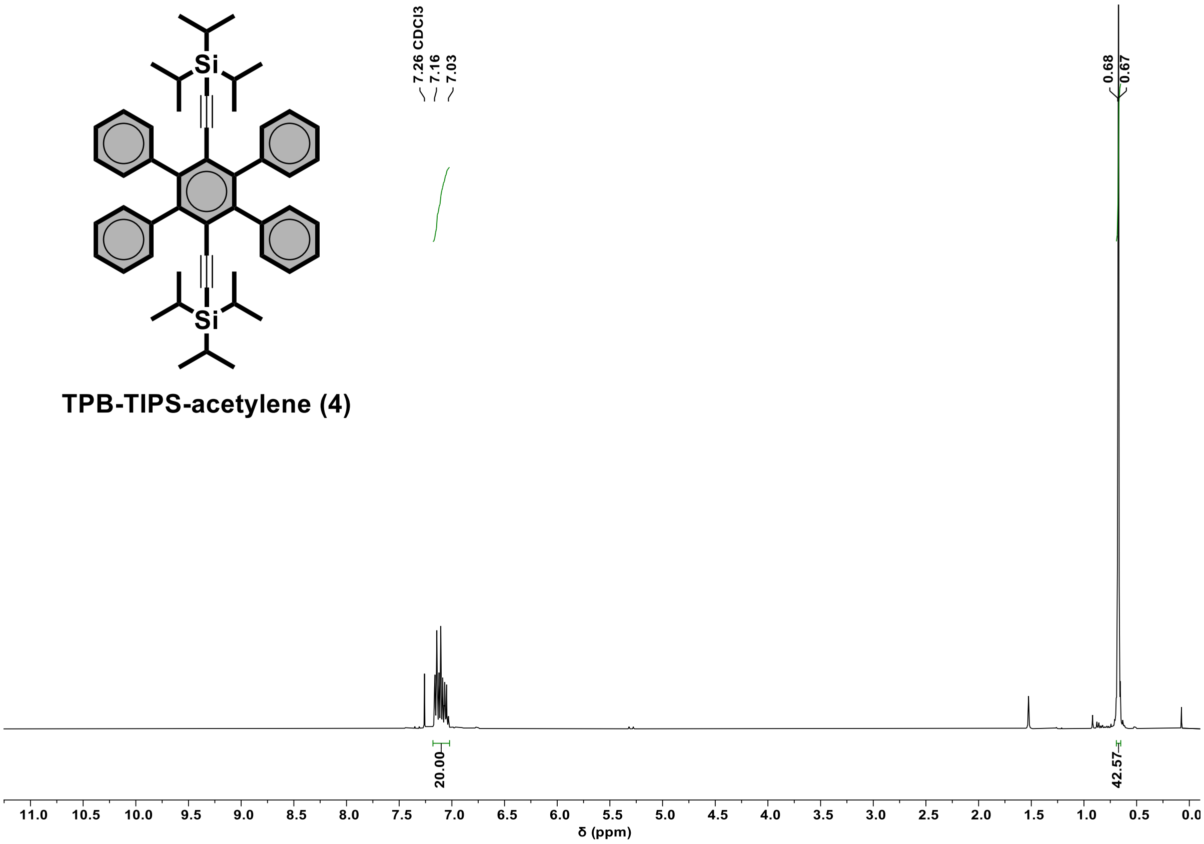

Figure S4: ^{1}H spectrum ($CDCl_3$, 400 MHz) of **TPB-TIPS-acetylene (4)**.

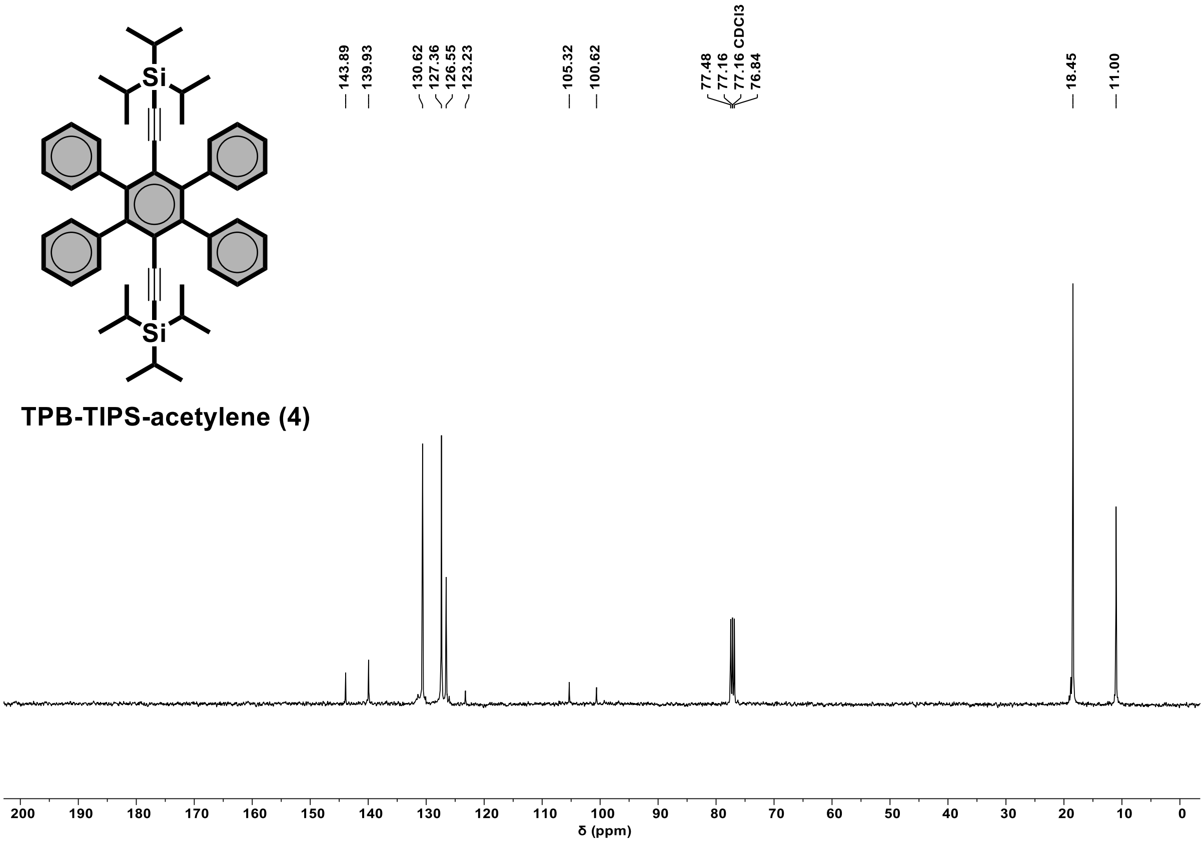

Figure S5: ^{13}C spectrum ($CDCl_3$, 101 MHz) of **TPB-TIPS-acetylene (4)**.

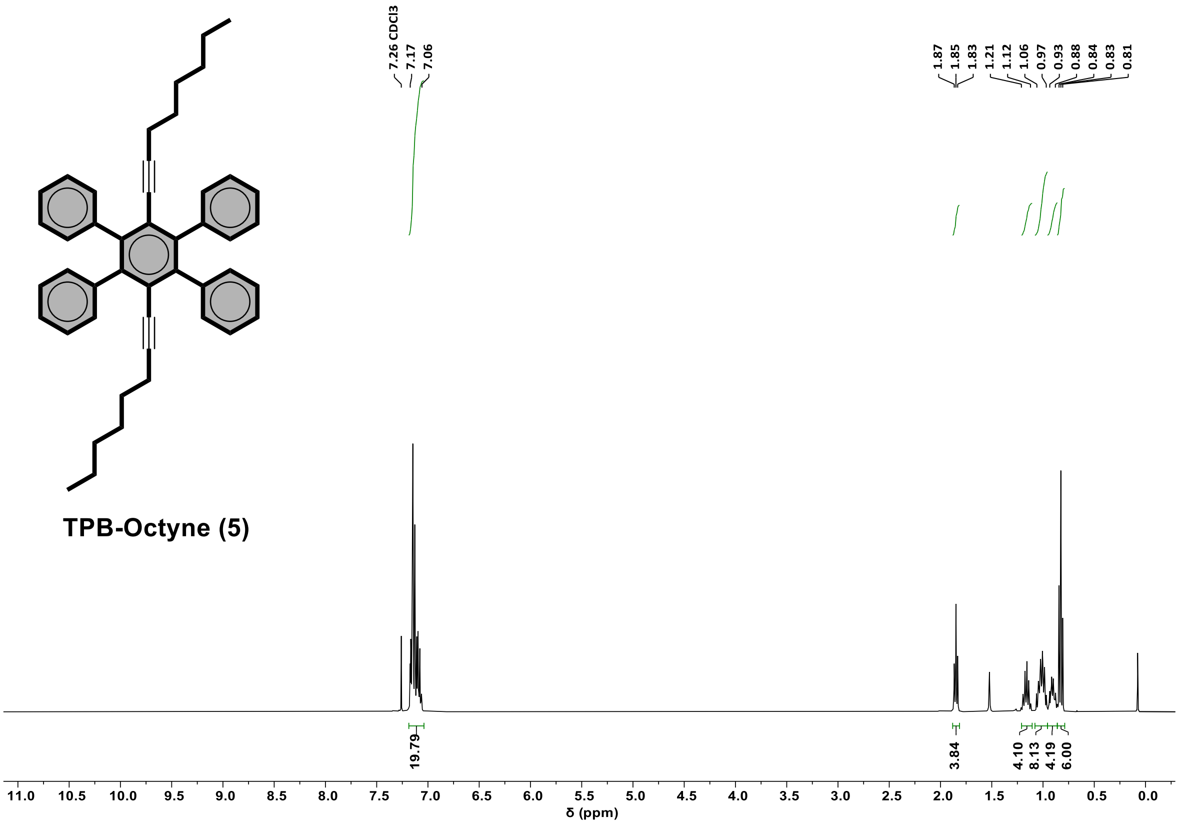

Figure S6: ^{1}H spectrum ($CDCl_3$, 400 MHz) of **TPB-Octyne (5)**.

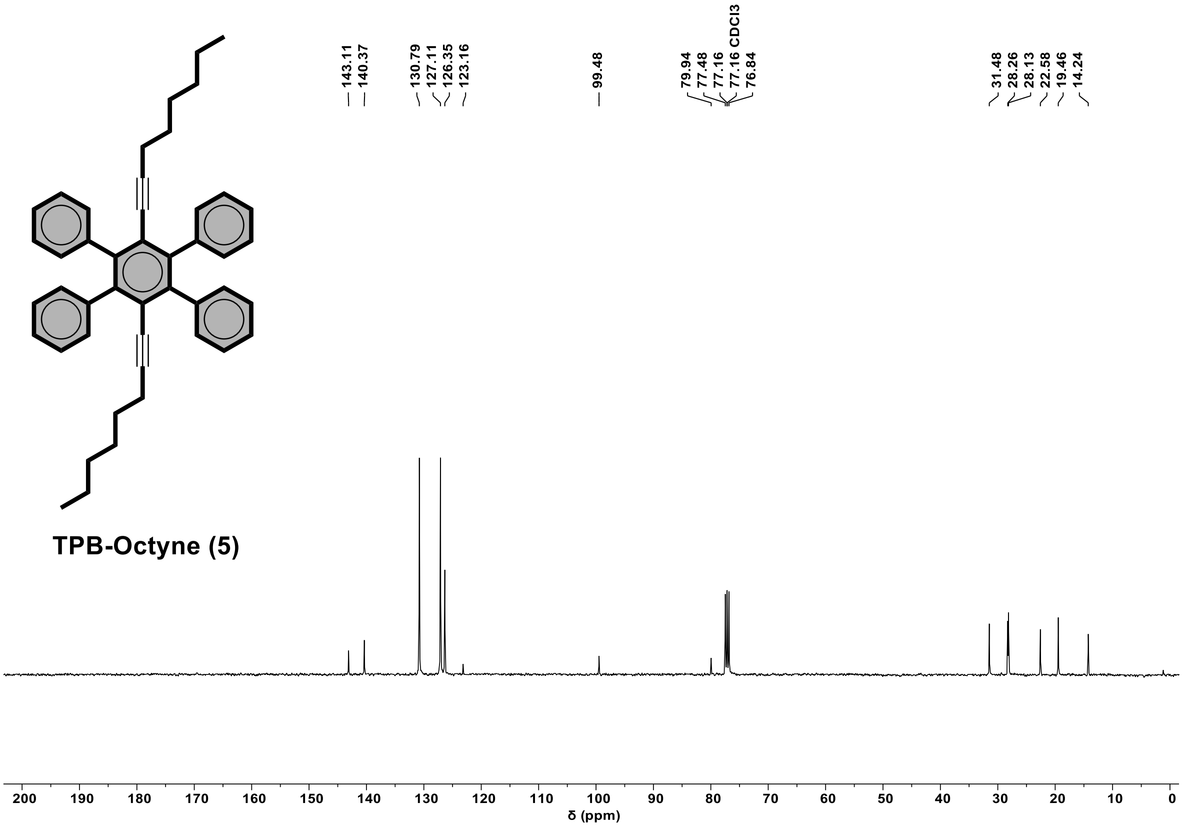

Figure S7: ^{13}C spectrum ($CDCl_3$, 101 MHz) of **TPB-Octyne (5)**.

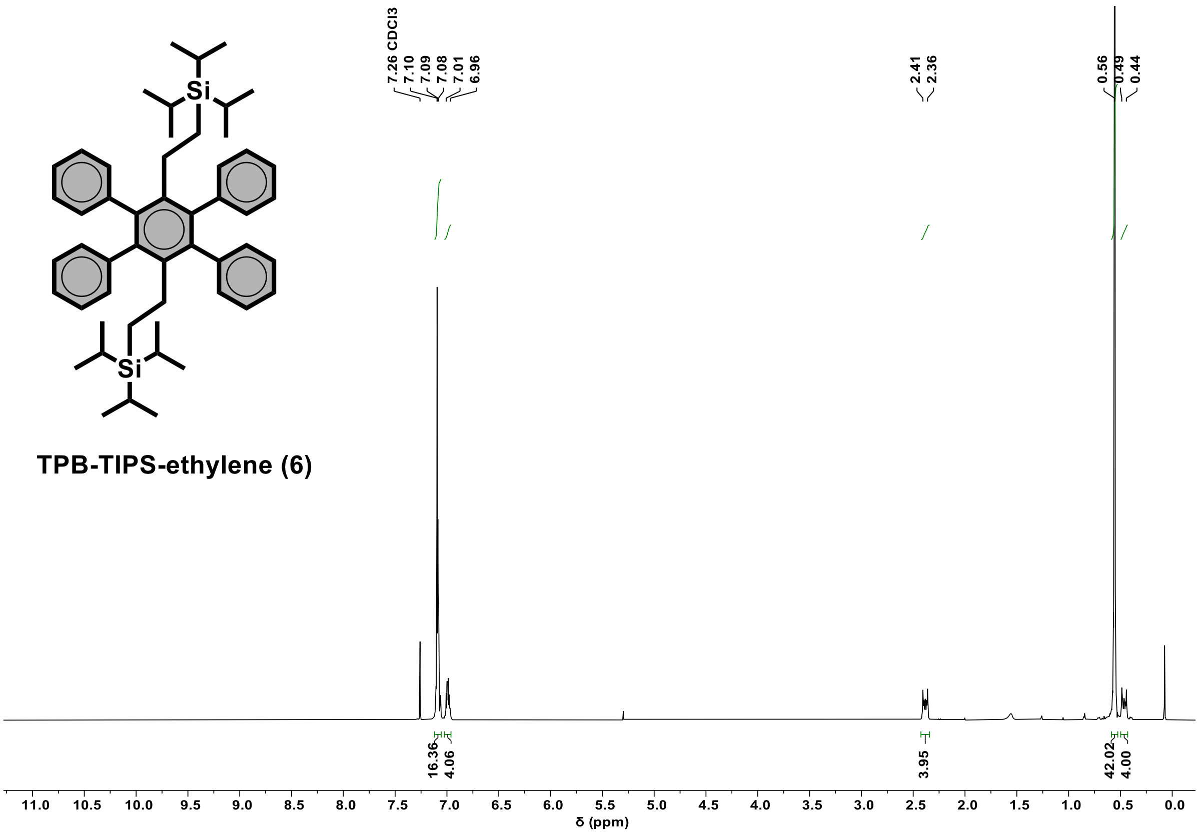

Figure S8: ^{1}H spectrum ($CDCl_3$, 400 MHz) of **TPB-TIPS-ethylene (6)**.

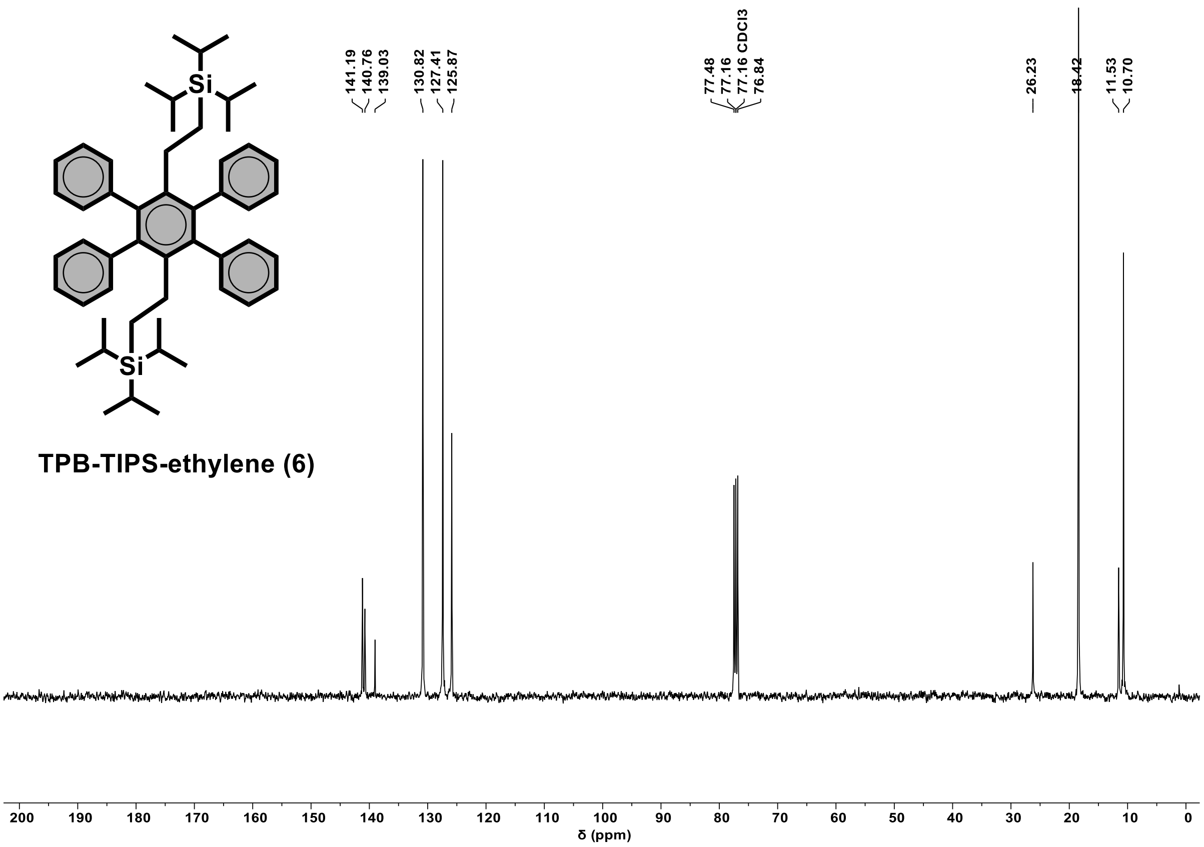

Figure S9: ^{13}C spectrum ($CDCl_3$, 101 MHz) of **TPB-TIPS-ethylene (6)**.

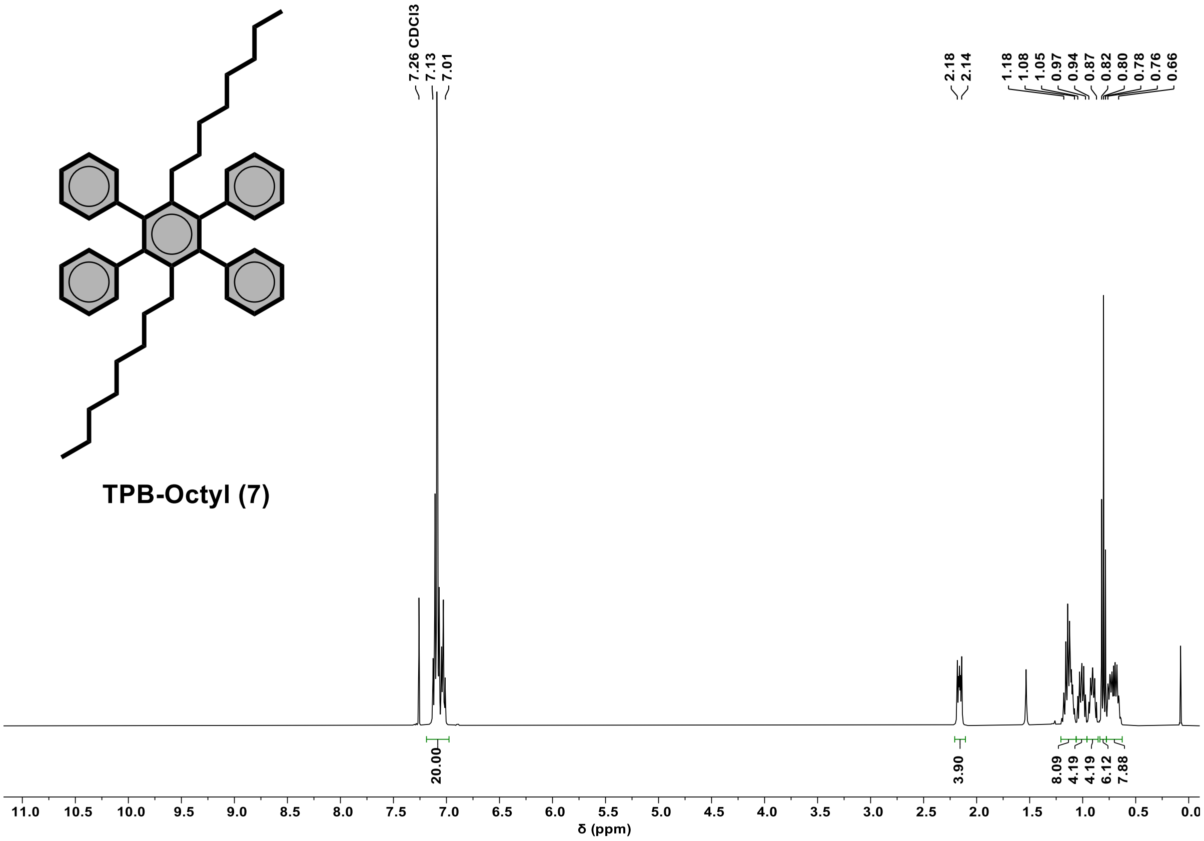

Figure S10: ^{1}H spectrum ($CDCl_3$, 400 MHz) of **TPB-Octyl (7)**.

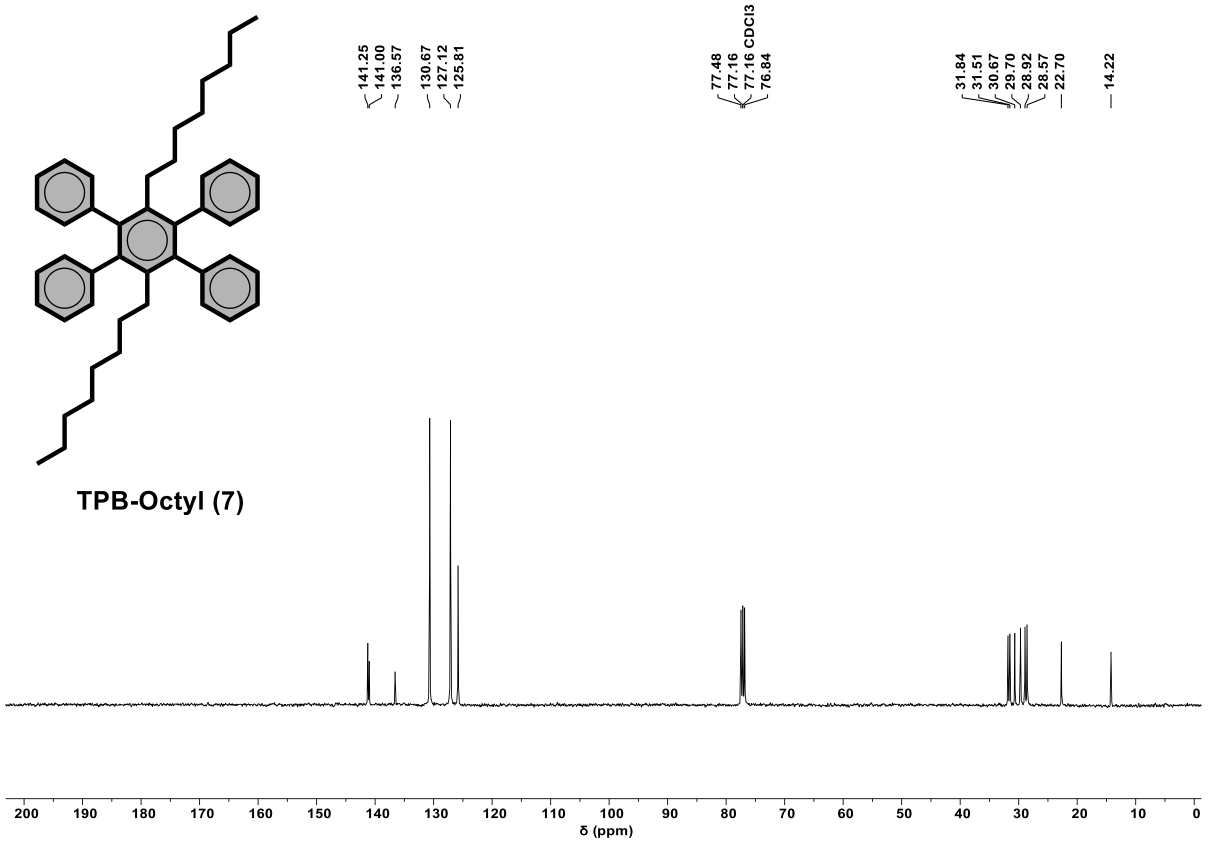

Figure S11: ^{13}C spectrum ($CDCl_3$, 101 MHz) of **TPB-Octyl (7)**.

MS spectra

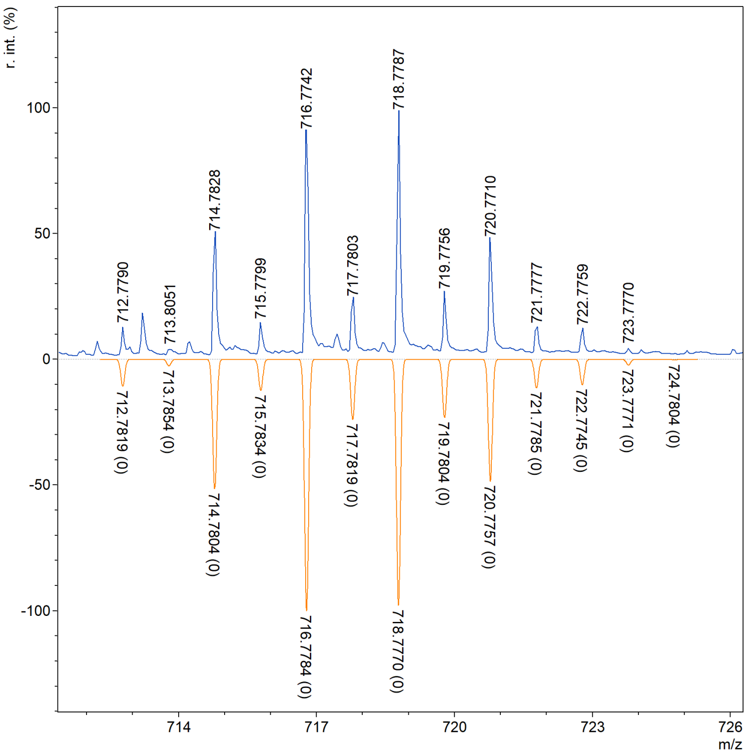

Figure S12: Experimental (top) and Simulated (bottom) MALDI-TOF spectrum of **3**. Matrix: 10 mg/mL DCTB + 0,1 mg/mL AgTFA in THF.

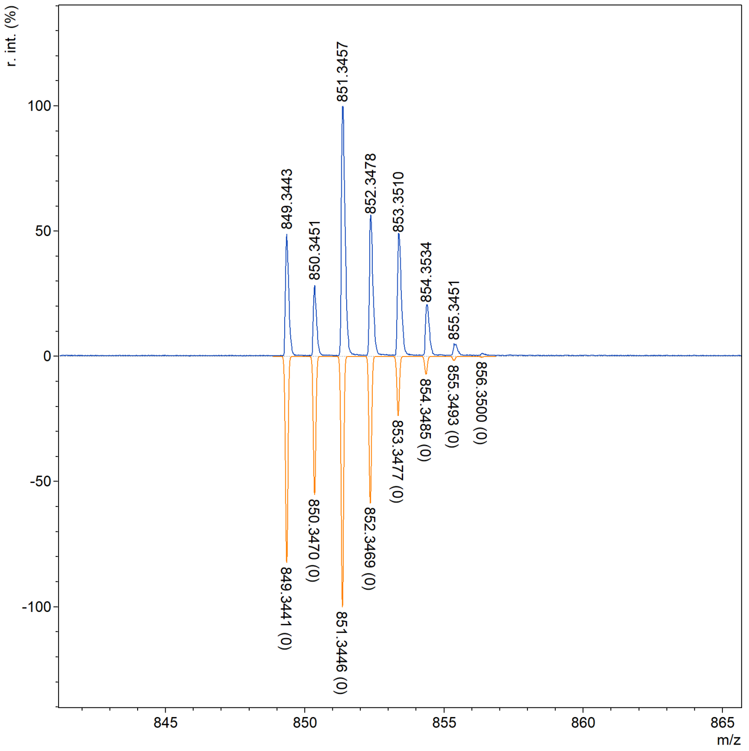

Figure S13: Experimental (top) and Simulated (bottom) MALDI-TOF spectrum of **TPB-TIPS-acetylene (4)**. Matrix: 10 mg/mL DCTB + 0,1 mg/mL AgTFA in THF.

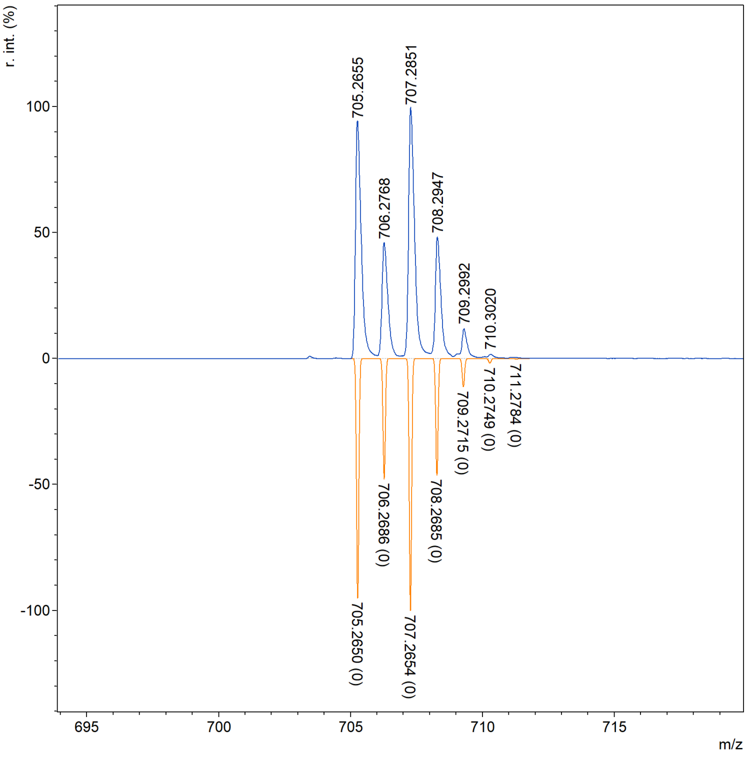

Figure S14: Experimental (top) and Simulated (bottom) MALDI-TOF spectrum of **TPB-Octyne (5)**. Matrix: 10 mg/mL DCTB + 0,1 mg/mL AgTFA in THF.

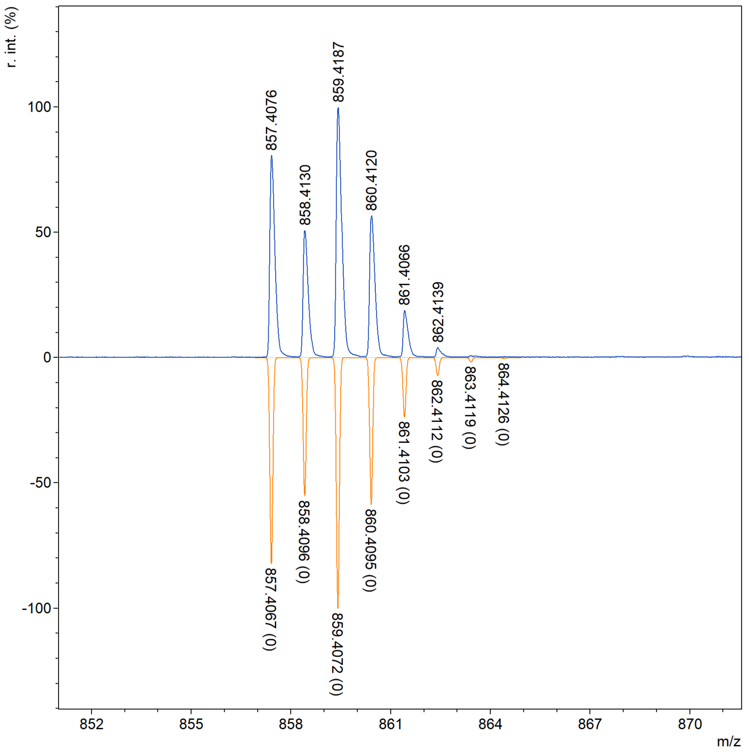

Figure S15: Experimental (top) and Simulated (bottom) MALDI-TOF spectrum of **TPB-TIPS-ethylene (6)**. Matrix: 10 mg/mL DCTB + 0,1 mg/mL AgTFA in THF.

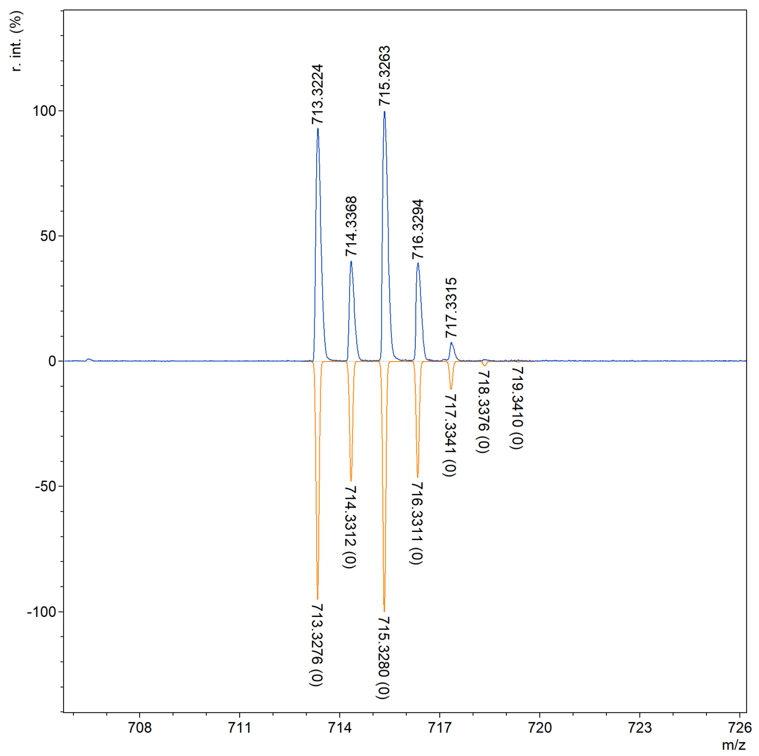

Figure S16: Experimental (top) and Simulated (bottom) MALDI-TOF spectrum of **TPB-Octyl (7)**. Matrix: 10 mg/mL DCTB + 0,1 mg/mL AgTFA in THF.

IR spectra

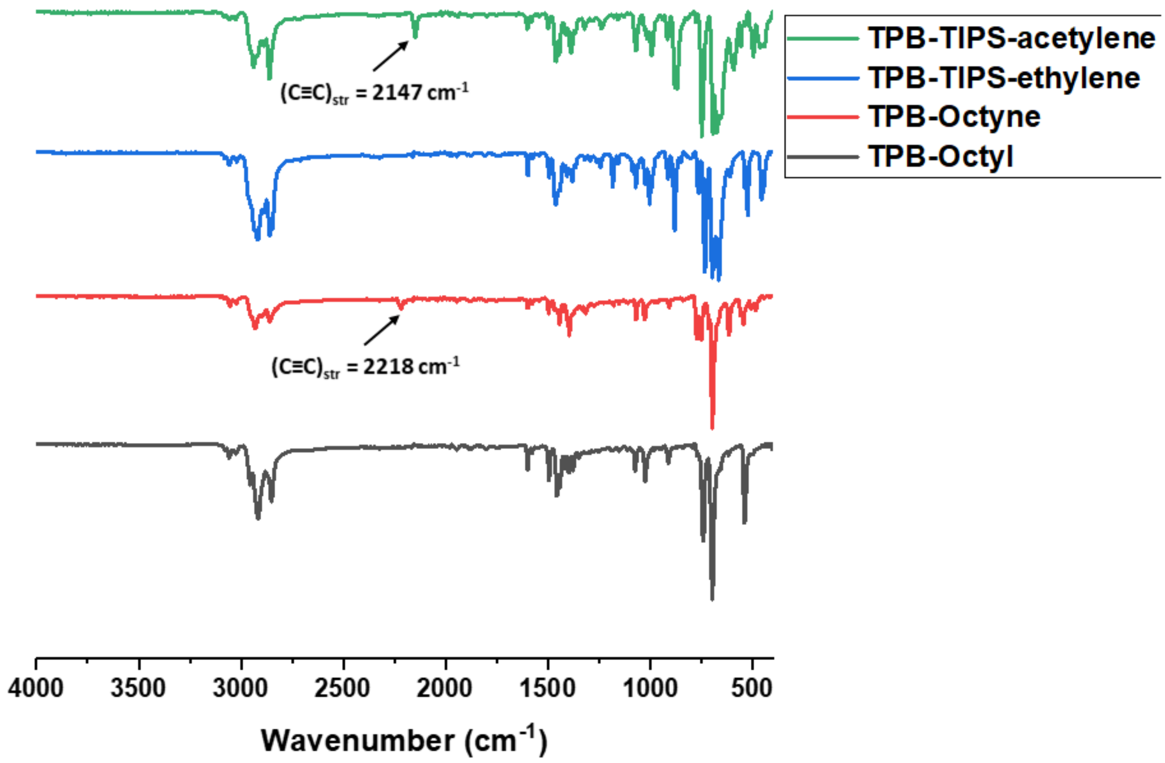

Figure S17: FTIR spectra of **TPB-TIPS-acetylene (4)** (green), **TPB-TIPS-ethylene (6)** (blue), **TPB-Octyne (5)** (red), and **TPB-Octyl (7)** (black).

Emission and Excitation spectra in pure THF

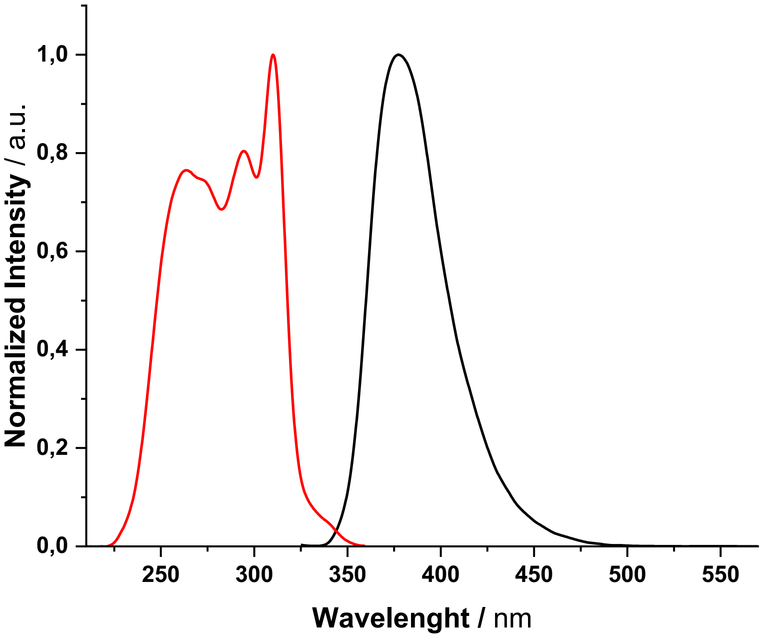

Figure S18: Emission (black, λ_{exc} = 315 nm) and Excitation (red, λ_{em} = 380 nm) spectra of **TPB-TIPS-acetylene (4)** in pure THF.

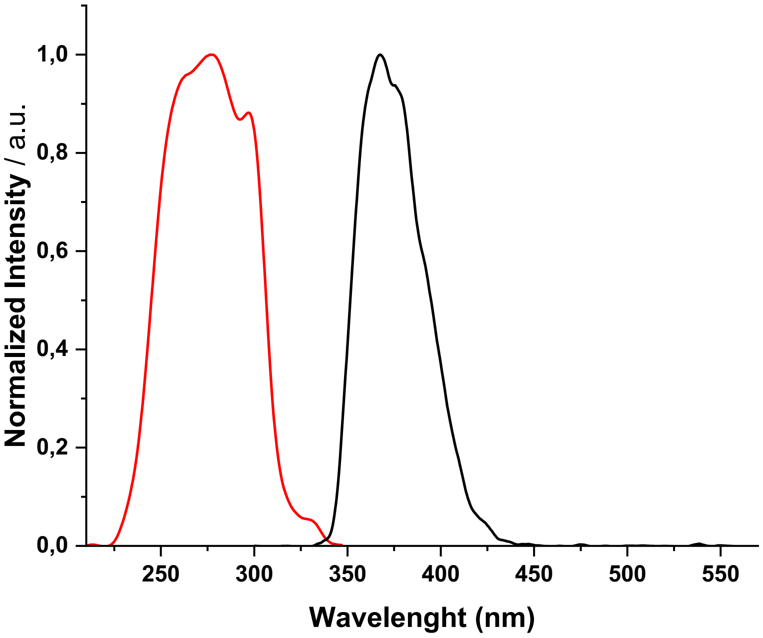

Figure S19: Emission (black, λ_{exc} = 297 nm) and Excitation (red, λ_{em} = 368 nm) spectra of **TPB-Octyne (5)** in THF.

Aggregation Induced Emission

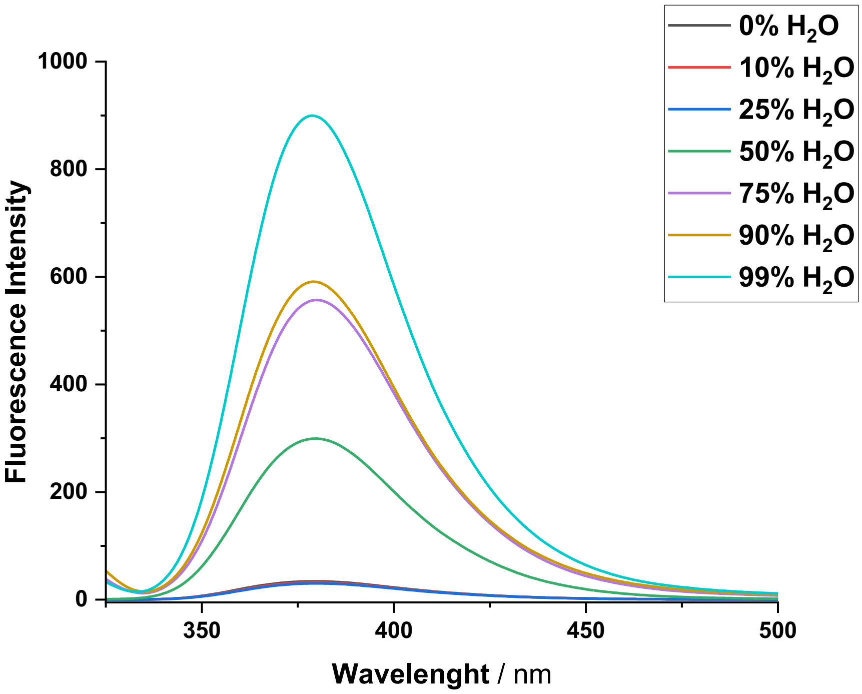

Figure S20: Emission spectra of equimolar solutions of **TPB-TIPS-acetylene (4)** with different percentages of H_2O in THF (λ_{exc} = 315 nm).

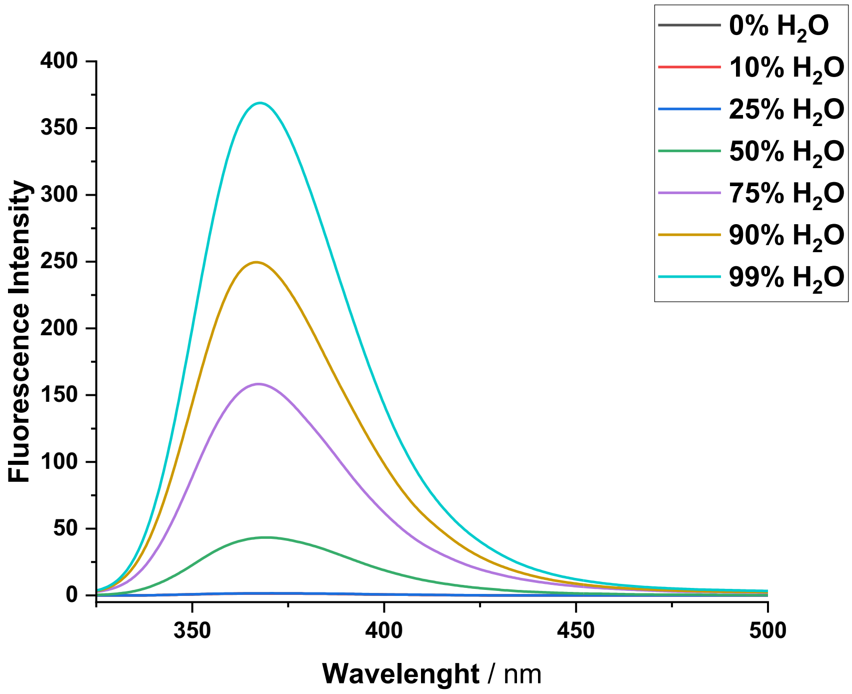

Figure S21: Emission spectra of equimolar solutions of **TPB-Octyne (5)** with different percentages of H_2O in THF (λ_{exc} = 297 nm).

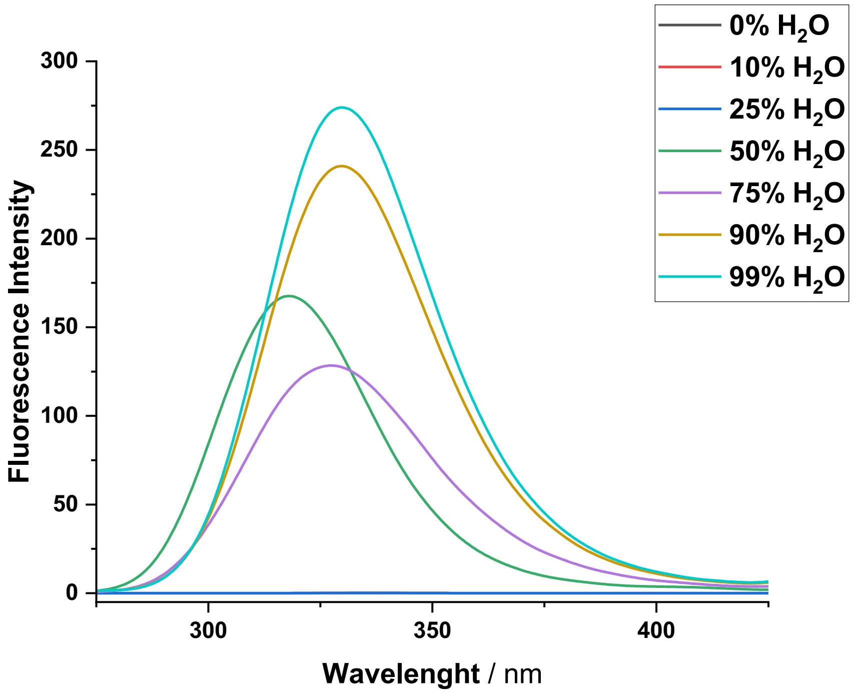

Figure S22: Emission spectra of equimolar solutions of **TPB-TIPS-ethylene (6)** with different percentages of H_2O in THF (λ_{exc} = 232 nm).

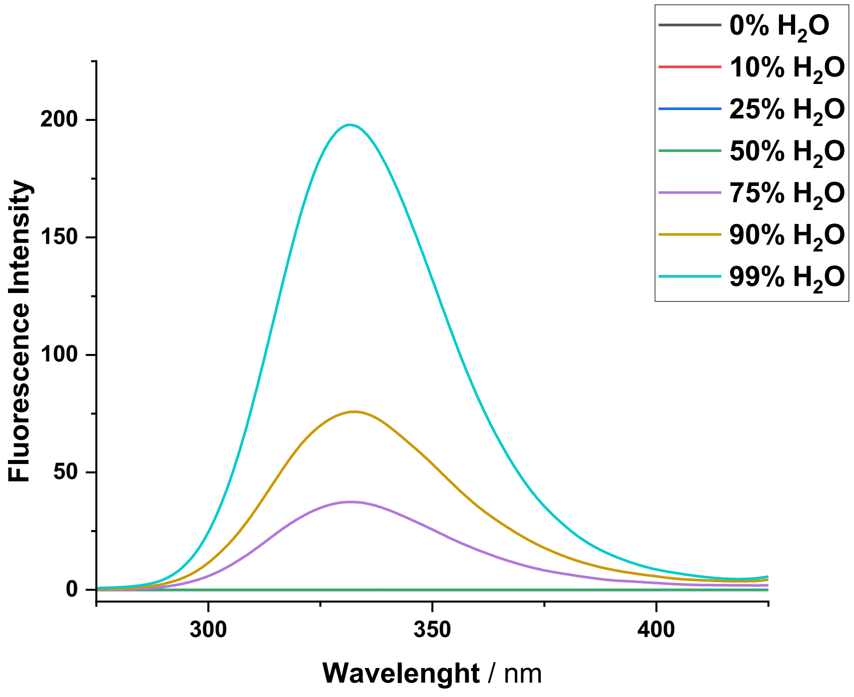

Figure S23: Emission spectra of equimolar solutions of **TPB-Octyl (7)** with different percentages of H_2O in THF (λ_{exc} = 230 nm).

Electrochemical characterization

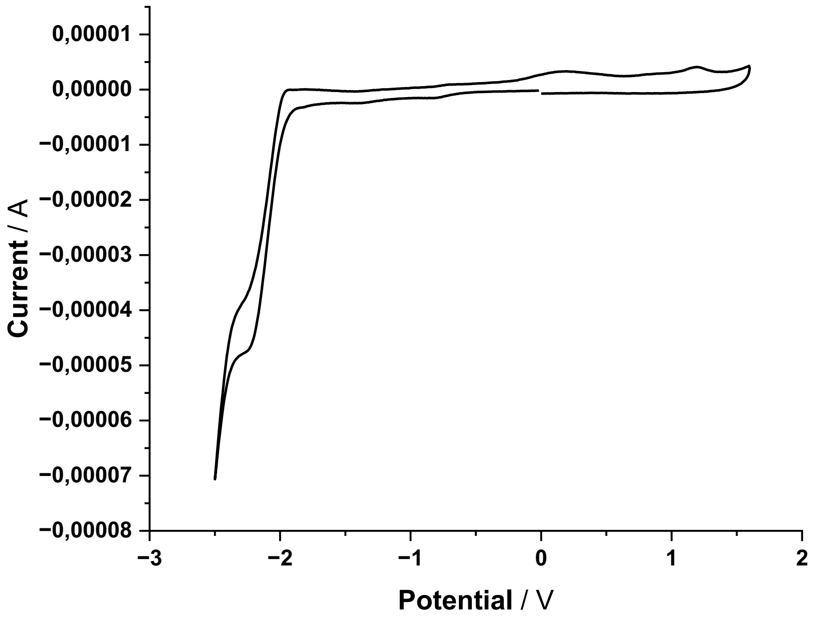

Figure S24: Cyclic voltammograms (IUPAC convention) of **TPB-TIPS-acetylene (4)** in n-Bu_4NPF_6/THF. Intial potential 0 V, reductive direction of initial scan.

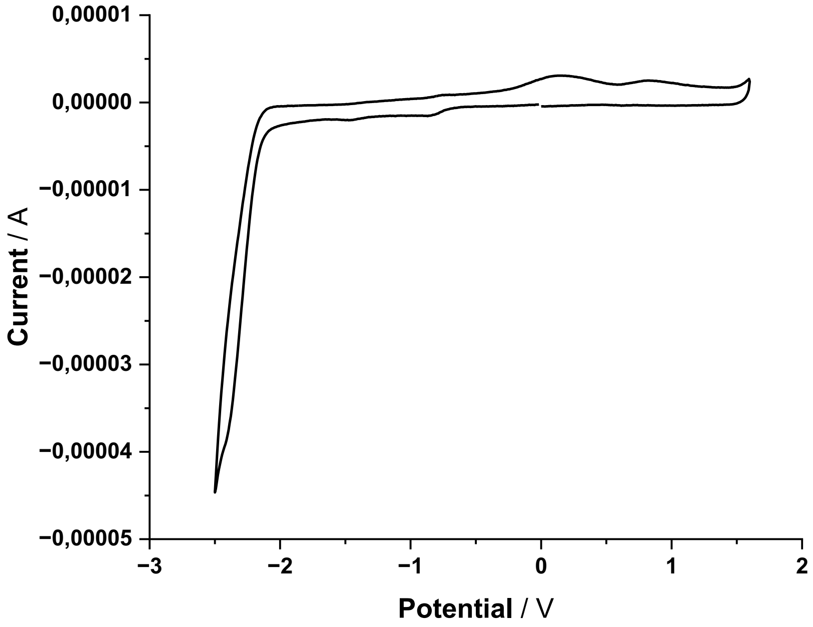

Figure S25: Cyclic voltammograms (IUPAC convention) of **TPB-Octyne (5)** in n-Bu_4NPF_6/THF. Intial potential 0 V, reductive direction of initial scan.

Table S1: Electronic properties of **TPB-TIPS-acetylene**, **TPB-Octyne**, **TPB-TIPS-ethylene**, and **TPB-Octyl**.

	**TPB-TIPS-acetylene (4)**	**TPB-Octyne (5)**	**TPB-TIPS-ethylene (6)**	**TPB-Octyl (7)**
$\mathbf{E_{onset}(red)_1}$ /V vs Fc^+/Fc [a]	-2.65	-2.82	N/A	N/A
$\mathbf{E_{LUMO}}$ /eV [b]	-2.45	-2.28	N/A	N/A
$\boldsymbol{\lambda_{onset}}$ /nm [c]	320	310	255	255
$\mathbf{E_g}$ /eV [d]	3.9	4.0	4.9	4.9

[a] Values determined at the onset of the first electrochemical reduction event. No events were observable for the alkane derivatives TPB-TIPS-ethylene and TPB-Octyl.
[b] LUMO level energies, approximated from the electrochemical analysis, from the onset potential of the first reduction event, assuming E_{Fc} = -5.1 eV for ferrocene $\mathbf{E_{LUMO}} = \mathbf{E_{Fc}} - \mathbf{E_{onset}(red)_1}$, units for each value as indicated in the table.
[c] Values determined at the onset of the lowest energy band observable on the absorption spectra in solution in THF.
[d] Optical bandgap energies, determined from the onset values of the lowest energy absorption band $E_g = 1240/\lambda_{onset}$, units as indicated in the table.

References

1. Aminabhavi, T.M.; Gopalakrishna, B. Density, Viscosity, Refractive Index, and Speed of Sound in Aqueous Mixtures of N,N-Dimethylformamide, Dimethyl Sulfoxide, N,N′-Dimethylacetamide, Acetonitrile, Ethylene Glycol, Diethylene Glycol, 1,4-Dioxane, Tetrahydrofuran, 2-Methoxyethanol, and 2-Ethoxyethanol at 298.15 K. *J. Chem. Eng. Data* **1995**, 40, 856–861.

2. VanVeller, B.; Miki, K.; Swager, T. M. Rigid Hydrophilic Structures for Improved Properties of Conjugated Polymers and Nitrotyrosine Sensing in Water. *Org. Lett.* **2010**, 12, 1292–1295.
